# Insights into the Initiation of JC Virus DNA Replication Derived from the Crystal Structure of the T-Antigen Origin Binding Domain

**DOI:** 10.1371/journal.ppat.1003966

**Published:** 2014-02-20

**Authors:** Gretchen Meinke, Paul J. Phelan, Radha Kalekar, Jong Shin, Jacques Archambault, Andrew Bohm, Peter A. Bullock

**Affiliations:** 1 Department of Developmental, Molecular and Chemical Biology, Tufts University School of Medicine, Boston, Massachusetts, United States of America; 2 Laboratory of Molecular Virology, Institut de Recherches Cliniques de Montreal, Montreal, Quebec, Canada; University of Michigan, United States of America

## Abstract

JC virus is a member of the Polyomavirus family of DNA tumor viruses and the causative agent of progressive multifocal leukoencephalopathy (PML). PML is a disease that occurs primarily in people who are immunocompromised and is usually fatal. As with other Polyomavirus family members, the replication of JC virus (JCV) DNA is dependent upon the virally encoded protein T-antigen. To further our understanding of JCV replication, we have determined the crystal structure of the origin-binding domain (OBD) of JCV T-antigen. This structure provides the first molecular understanding of JCV T-ag replication functions; for example, it suggests how the JCV T-ag OBD site-specifically binds to the major groove of GAGGC sequences in the origin. Furthermore, these studies suggest how the JCV OBDs interact during subsequent oligomerization events. We also report that the OBD contains a novel “pocket”; which sequesters the A1 & B2 loops of neighboring molecules. Mutagenesis of a residue in the pocket associated with the JCV T-ag OBD interfered with viral replication. Finally, we report that relative to the SV40 OBD, the surface of the JCV OBD contains one hemisphere that is highly conserved and one that is highly variable.

## Author Summary

Polyomaviruses have been invaluable tools for biomedical research into basic cellular processes. It is becoming increasingly clear, however, that members of this family are also involved in human diseases, particularly among the immunocompromised and the elderly. The subject of this study, the JC virus (JCV), is a member of this family and the causative agent of a brain disease termed Progressive Multifocal Leukoencephalopathy (PML), a disease that is often fatal and for which there is no cure. Herein we present the high-resolution crystal structure of the origin binding domain (OBD) from the JCV initiator protein large T-antigen. Furthermore, we propose a molecular model for the oligomerization of the JCV T-antigen OBD that is based upon the crystal structure. We also report a novel pocket that modeling studies suggest is available when the OBD is site-specifically bound to DNA and therefore may represent a possible starting point for structure-based drug design.

## Introduction

There are now twelve known human polyomavirus members (e.g., [1], [2]) and particularly for immuno-compromised individuals, there is an increasing association between these viruses and human diseases (reviewed in [3], [4], [5]). For example, JC virus (JCV) is the causative agent of Progressive Multifocal Leukoencephalopathy ((PML); reviewed in [6], [7], [8]); a demyelinating disease of the central nervous system [9], [10]. JCV is also a major opportunistic infection associated with acquired immunodeficiency syndrome [11], occurring in up to 5% of AIDS patients [12]. Further interest in JCV, which is present in approximately 50% of the general population [13], stems from the fact that a promising new treatment of multiple sclerosis (the monoclonal antibody Tysabri) is known to be associated with the induction of PML (reviewed in [8], [14], [15]). Studies have also suggested a possible association between infection with JCV and human brain and non-central nervous system tumors [16], [17]. Unfortunately, there is no specific treatment for JCV.

Central to the JCV life cycle is the replication of its genome. The JCV origin of replication has been the topic of numerous studies (e.g., [18], [19], [20], [21], [22]). The interactions between the origin with the viral initiator, large T-antigen (T-ag), has also been explored (e.g., [23], [24]). The T-antigens encoded by polyomaviruses are multi-domain, multifunctional proteins (reviewed in [25], [26]) that form hexamers and double hexamers at origins of replication (reviewed in [27]). Assays designed to monitor T-ag dependent JCV replication have been reported (e.g., [18], [28]), including a cell free replication system [29]. However, theories regarding how JCV replication takes place are largely based on studies of the replication of Simian Virus 40 (SV40) (reviewed in [26], [30], [31], [32]). For example, an in depth understanding of the enzymology of SV40 DNA replication was obtained following many elegant studies (reviewed in [32], [33], [34]). Related studies have focused on the roles played by the T-ag during SV40 replication (reviewed in [25], [27], [35], [36]). Our laboratories have focused on the multiple roles played by the central origin-binding domain (OBD) of the SV40 T-ag during viral replication (reviewed in [37], [38]). Functions of the OBD include site-specific binding to GAGGC sequences in the origin ([39], [40]), promoting oligomerization of T-ag (e.g., via the B3 motif [41], [42]), melting of the central region of the core origin [43], binding to ssDNA at replication forks [37], [44], [45] and recruiting cellular initiation factors (e.g., [46]).

Structural studies of T-ag have provided critical insights into how this single domain can engage in so many activities (reviewed in [37]). For example, structures of the SV40 T-ag OBD established how the A1 & B2 loops in the OBD bind site-specifically to the GAGGC repeats in the central region of the viral origin (i.e. Site II) [42], [47], [48], [49]. They also established how the same A1 & B2 loops engage other DNA structures (e.g., duplex DNA in a non-sequence specific manner [49] and ssDNA [44], [45]). Crystallography studies also established that the SV40 T-ag OBDs can bind to a fork like DNA structure [45]. The latter observation was one reason for suggesting that the SV40 T-ag OBD is eventually positioned at the replication forks (reviewed in [37]). The structures of additional domains of T-ag have provided many additional insights into the interactions needed to initiate viral DNA replication (e.g., [47], [50], [51], [52]). For example, structures of the C-terminal helicase domain have greatly increased our understanding of how hexameric helicases catalyze DNA replication (reviewed in [53]) and how the helicase and OBDs work together to interact with ds DNA [47].

The initiation of JCV DNA replication is a central event during the viral life cycle [24]. The shared nucleotide sequence identity between the T-ag genes of SV40 and JCV is 71% [54]. Therefore, it is perhaps not surprising that SV40 T-ag recognizes and binds the JCV origin both *in vivo* and *in vitro* [24], [55], [56], [57]. Studies show that the converse is not true; that is JCV T-ag is inefficient at promoting replication of an SV40 origin-containing plasmid [24]. Thus, while T-ag and the other proteins encoded by these viruses are highly homologous, they likely contain subtle but important structural differences. To examine these issues, we pursued structural and biophysical studies of the JCV T-ag OBD. The results from these studies suggest how the JCV T-ag OBD binds to the viral origin and its subsequent roles in oligomerization events. They also demonstrate that the JCV OBD contains a pocket that has not been described in previous structures of the polyomavirus OBDs. Collectively, these findings provide a preliminary molecular understanding of the initiation of JC virus replication.

## Materials and Methods

### I. Molecular Biology Techniques

#### 1. Sub-cloning of plasmids

A plasmid encoding the cDNA for JCV T-ag was obtained from Dr. H.P. Nasheuer. DNA encoding just the JCV T-ag was obtained by PCR and then subcloned into plasmid pCMVneo via Gibson Assembly (New England Biolabs). The resulting plasmid was termed pCMV JC T-ag. (The JCV T-ag gene was placed in the same position in pCMV as the gene encoding the SV40 T-ag in the previously described pCMV SV40-T-ag plasmid [58]). Sequences encoding the JCV T-ag origin-binding domain (OBD; residues 132–261) were isolated from plasmid pCMV JCT by PCR. Using Gibson Assembly, the OBD encoding sequences were subcloned into plasmid pGEX 1λT. The resulting plasmid, which expresses the OBD as an N-terminal GST fusion, was termed pGEX1λT JC-OBD.

#### 2. Overexpression and purification of JCV OBD (amino acids 132–261)

The plasmid pGEX1λT JC-OBD was transformed into BL21 (DE3) cells. Six liters of 2×YT medium was supplemented with ampicillin to 100 ug/ml and inoculated with an overnight culture (10 ml/liter (1∶100)). The cells were grown at 37°C with shaking until the A600 was 0.7 to 0.8. At that point the temperature was dropped to 28°C and IPTG was added to a final 0.1 mM. The cells were harvested 16 hours later and spun down at 7000 g for 20 min. Each 1 liter pellet was re-suspended in ∼20 ml of lysis buffer (1× PBS, 0.4 M NaCl, 10% glycerol, 1%NP- 40, 1 mM PMSF, 1 mM EDTA and 1× protease cocktail (0.2 mM AEBSF, 20 uM Bestatin, 3 uM E-64, 3 uM Pepstatin A)). The cells were lysed by passing them four times through a microfluidizer. The lysate was ultracentrifuged at 125,000 g for 30 min. and the supernatant was loaded onto a pre-equilibrated GSTPrep FF 16/10 glutathione sepharose column (GE, Inc). The column was washed with lysis buffer until the absorbance @ 280 was <0.1, followed by 100 ml of wash buffer (1×PBS, 10% glycerol and 0.1% *B*-mercaptoethanol). The GST-OBD fusion protein was eluted in elution buffer (50 mM Tris pH 8.0, 0.15 M NaCl, 10% Glycerol and 10 mM glutathione pH 8.0). Thrombin was added (1∶500 thrombin∶protein) and the reaction was dialyzed overnight against 2 liters dialysis buffer (20 mM Tris pH 8.0, 50 mM NaCl and 10% Glycerol). The dialysate was spun down and to remove precipitated protein, filtered through a 0.22 um filter. The supernatant was loaded onto pre-equilibrated 25 ml Source 15Q and Source 15S columns (GE) attached in tandem. After washing the columns, the Source 15Q column was disconnected and a gradient of 0.05–1 M NaCl was applied to the Source 15S column; protein containing fractions were collected and analyzed by SDS-PAGE. The fractions containing the isolated JCV OBD were pooled, concentrated and loaded onto a Superdex-200 26/60 column equilibrated in storage buffer (20 mM Tris pH 8.0, 50 mM NaCl, 10% glycerol, 1 mM EDTA, 0.1 mM PMSF and 5 mM DTT). The protein containing fractions were analyzed by SDS-PAGE, pooled and concentrated to ∼10 mg/ml. The purified protein was aliquoted, quick frozen in liquid nitrogen and stored at −80°C in storage buffer. The protocol yielded ∼10–12 mg purified JCV OBD/L of culture.

### II. Biophysical Techniques

#### 1. Crystallization

Crystallization trials of the JCV OBD using the Qiagen and Micro lytics MCSG crystallization screens resulted in three separate crystal forms. Crystal form 1 grew in hanging drops at 18 C by vapor diffusion over a 1 mL reservoir in a Linbro plate (Hampton Research Inc) upon mixing 1 µl of the protein (at 10 mg/ml in storage buffer) with 1 µl of the reservoir solution (0.1 M sodium citrate pH 5.6, 30% PEG 3350). Rod-shaped crystals appeared in a few days and were then harvested by first transferring them to a cryogenic solution (0.1 M sodium citrate pH 5.6, 35% PEG 3350, 15% glycerol) using a cryo-loop and then flash-freezing the loop into liquid nitrogen until ready for x-ray data collection. Crystal form 2 grew at 4 C from sitting drops in a 96 well tray (CrystalQuick plates from Hampton Research, Inc) by vapor diffusion over 50 µl reservoir solution upon mixing 1 µl of the protein with 1 µl of the reservoir solution (0.1 M Tris pH 8.5, 0.2 M LiSO_4_, 30% PEG 4000). They were smaller than crystal form 1, brick shaped and appeared in a few days. These crystals were transferred to a cryogenic solution (0.05 M Tris pH 8.5, 0.15 M LiSO_4_, 35% PEG 4000, 10% glycerol), and flash-cooled in liquid nitrogen for storage until ready for x-ray data collection. Crystal form 3 was grown at 4 C, upon mixing 1 µl of the protein with 1 µl of the reservoir solution (0.19 M sodium tartrate, 19% PEG 3350) in sitting drops by vapor diffusion over 50 µl reservoir solution. These crystals were morphologically similar to crystal form 1 and appeared in a few days. Crystals were transferred to a cryogenic solution (0.2 M sodium tartrate, 35% PEG 3350, 10% glycerol) and flash-cooled in liquid nitrogen for storage until ready for x-ray data collection.

#### 2. X-ray data collection and structure solution

All final high-resolution X-ray data were collected at 100 K at the NSLS Beamline X29 (Brookhaven National Laboratory, NY). The x-ray data were processed with HKL2000 [59]. Data sets of 1.64 Å, 2.6 Å, and 1.32 Å were collected for crystal form 1, form 2 and form 3, respectively. The details of the data collection and refinement are summarized in Table 1.

**Table 1:** X-ray data collection and refinement statistics of the JCV OBD.

	Crystal Form 1	Crystal Form 2	Crystal Form 3
**Crystal Data PDB ID**	**4LMD**	**4LIF**	**4NBP**
Wavelength (Å)	1.075	1.075	1.075
Space group	C2	I4_1_	P4_1_
Cell dimensions			
*a*, *b*, *c* (Å)	145.64,37.05,64.741	103.793,103.793,34.86	64.663,64.663,37.493
α, β, γ (°)	90.0,112.766,90.0	90.0, 90.0, 90.0	90.0, 90.0, 90.0
Resolution (Å)	50-1.64 (1.70-1.64)*	50-2.6 (2.69-2.60)	50-1.32 (1.47-1.32)
*R*_sym_ or *R*_merge_	8.4% (58.7%)	6.8% (98.4%)	9.3% (65.8%)
Mean *I*/σ*I*	27.44 (2.67)	37.6 (2.58)	29.6 (4.2)
Completeness(%)	98.7 (87.3)	100% (99.8%)	99.9% (99.4%)
Redundancy	7.8 (6.3)	14.7 (13.7)	16.0 (12.5)
**Refinement**			
Resolution (Å)	1.64	2.6	1.32
No. reflections	48003	10674	36943
*R*_work_/*R*_free_	16.7/20.2	18.4/21.8	16.79/17.98
No. atoms			
Protein	2215	1031	1089
Water	374	5	223
*B*-factors			
all	27.44	74.5	21.90
R.m.s. deviations			
Bond lengths (Å)	0.014	0.007	0.057
Bond angles (°)	1.53	0.98	4.40

* Values in parentheses are for highest-resolution shell.

The space group of crystal form 1 was determined to be C2. The structure of crystal form 1 was solved by molecular replacement (MR) using the program PHASER [60] available within the CCP4 suite [61]. The program PyMOD [62] was used to generate a search model from the coordinates of the SV40 T-antigen OBD crystal structure (PDB ID = 2FUF) [63]. The program identified two copies of the domain in the asymmetric unit. The space group of crystal form 2 was determined to be I4_1_. Crystal form 2 was also solved by MR, using the same programs, but using as a search model a partially refined JCV OBD from crystal form 1. Crystal form 3 was determined to be space group P4_1_ having one molecule in the asymmetric unit and was also solved by MR. A similar refinement strategy was followed for each crystal form. The phases from the MR solutions were input to the program Arp-Warp [64] for automated structure building. Both the program Refmac5 [65], within the CCP4 suite, and the program Phenix [66] were used to refine the structure at different stages. The molecular graphics program Coot [67] was used for manual rebuilding between successive rounds of refinement. Crystal form 3 contained a covalently modified Lysine residue (Lys 168). The modified amino acid refinement parameters were generated using the program JLigand [68]. The refined coordinates and data for the three crystal forms have been deposited to the Protein Data Bank (PDB) and given the accession codes 4LMD, 4LIF and 4NBP respectively.

#### 3. Structural analyses and molecular visualization

Superposition of coordinates was carried out using the program SSM [69]. Analyses of protein structures was performed using the program PDBSUM [70]. PISA [71] was used to analyze protein-protein interfaces. Unless otherwise indicated, all molecular graphics images were generated using the program PyMOL [72]. Clustal Omega [73] was used to generate amino acid sequence alignments. Jalview [74] was used to visualize sequence alignments. The previously solved structures used in the molecular modeling discussed herein are the apo SV40 T-ag OBD and DNA bound SV40 T-ag OBD (RCSB PDB codes 2FUF, and 2NTC).

#### 4. Isothermal Titration Calorimetry (ITC)

ITC data were collected with a VP-ITC calorimeter (Microcal, Northampton, MA); the data were analyzed with Origin software provided by the manufacturer. The double stranded oligonucleotides used in these experiments are presented in the figure legend. Prior to the experiments, the dsDNA oligonucleotides and proteins were buffer-exchanged into 10 mM Sodium Phosphate, pH 7.0, 50 mM NaCl buffer using PD-10 columns (GE Healthcare). Protein and DNA concentrations were determined spectrophotometrically, using calculated extinction coefficients from the ProtParam web server, (http://ca.expasy.org/tools/protparam.html) and the IDTDNA website (http://biophysics.idtdna.com/UVSpectrum.html), respectively.

### III. JCV Replication Reactions

A luciferase based assay for studies of polyomavirus DNA replication was previously reported [75]. We developed a similar assay for measuring levels of JCV replication (unpublished) using the pCMV JC T-ag plasmid and a second plasmid containing the JCV origin of replication that was termed pJCV ori. Additional replication reactions were conducted with JCV T-ags containing point mutations introduced at selected residues using the QuikChange Kit ((Agilent); with oligonucleotides containing the desired mutation. Western blots, conducted with the Pab 416 antibody against T-ag (Santa Cruz Biotechnology), were used to determine whether a given point mutation disrupted T-ag's stability.

## Results

### I. The Structure of the JCV T-ag-OBD

**Figure 1:**
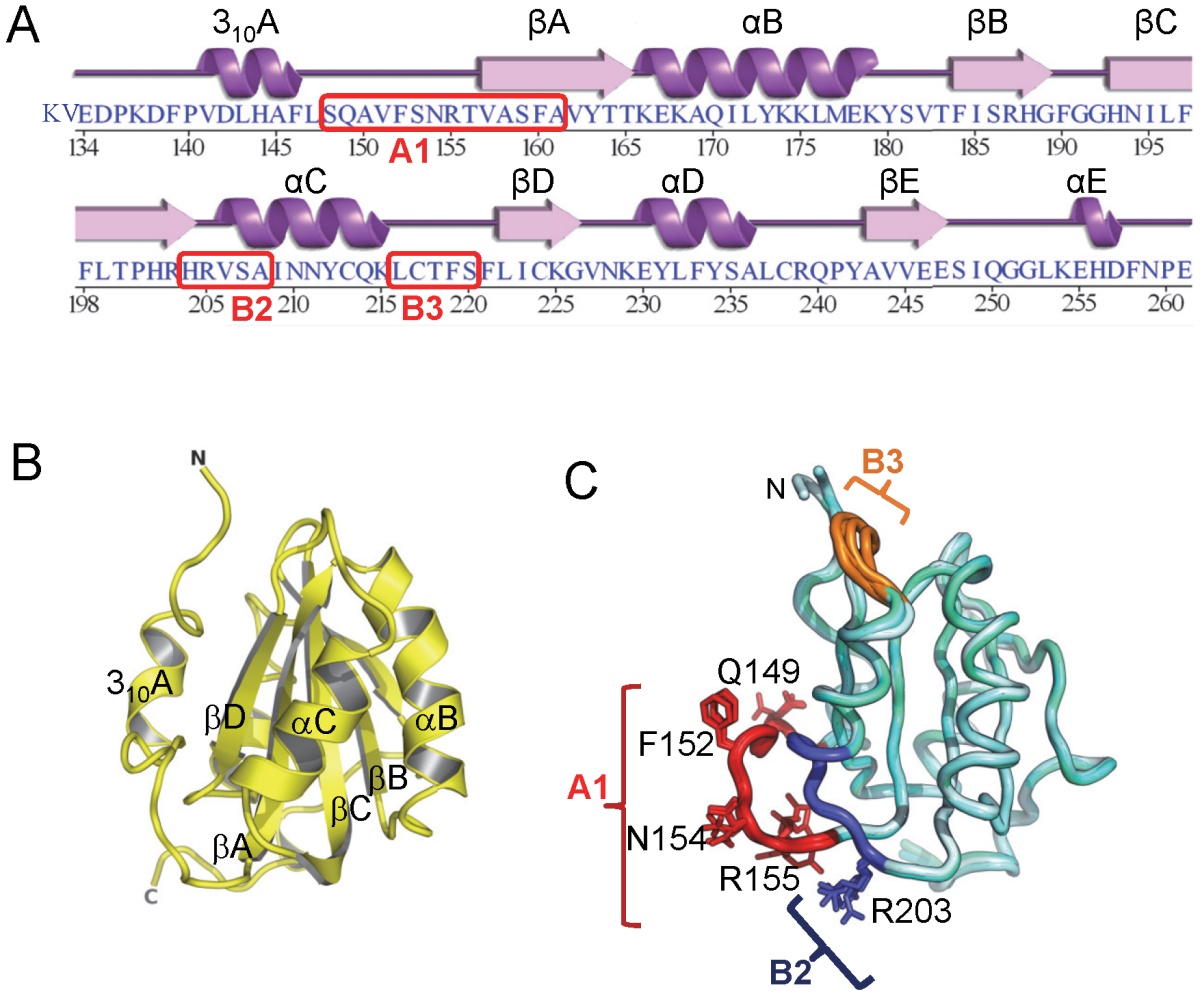
The structure of the JCV OBD. A . The amino acid sequence of the JCV T-ag origin binding domain (OBD). The associated secondary structures are presented above the primary sequence. The positions of the A1, B2 and B3 motifs are indicated. **B**. A ribbon diagram of the JCV OBD crystal structure. The individual beta strands and alpha helices are indicated, as are the N and C termini. The individual secondary elements were named as previously described for the SV40 OBD [48]. **C**. Superposition of all four structures of the JCV OBD (forms 1, 2 and 3), indicating where the DNA binding A1 and B2 loops are located along with the B3 motifs (in brown, blue and orange, respectively). Structurally, the most variable region in the OBD is the B3 motif.

The JCV OBD (residues 132–261 (Fig. 1A)) crystallized in three different forms that were termed form 1, form 2 and form 3 (Table 1). Form 1 has two molecules in the asymmetric unit cell, and together the three crystals provide four independent structures of the JCV OBD. The four structures are very similar; a superposition of the four JCV OBD structures revealed root mean-squared deviations (RMSDs) of less than 0.5 Å. Form 3, the highest resolution structure (1.32 Angstroms), is shown in Fig. 1B. The topology of the JCV OBD is a five-stranded antiparallel β-sheet sandwiched between two helices on either side (Figs. 1 A and B). A superposition of the four JCV OBDs structures onto the DNA-free SV40 OBD structure (the only other polyomavirus OBD to be solved in the absence of DNA [63]) revealed an additional low RMSD (between 0.85–0.88 Å over 121 Cα atoms).

JCV OBD region B3 (residues 216–220 [40]) is poorly ordered in crystal forms 1 and 2, but well ordered in form 3 (Fig. 1C). This loop is also poorly ordered in several of the SV40 OBD structures [45], [76], [77]. B3 is well ordered in form 3 because tartrate (a component in the crystallization mixture) modified lysine 168 in a manner analogous to lysine acetylation. The carboxyl groups of the tartrate stabilized the B3 residues via a series of backbone hydrogen bonds. There are no previous reports indicating that JCV T-ag Lys168 is acetylated and further studies are necessary to determine if the observed modification of Lys168 is functionally important.

**Figure 2:**
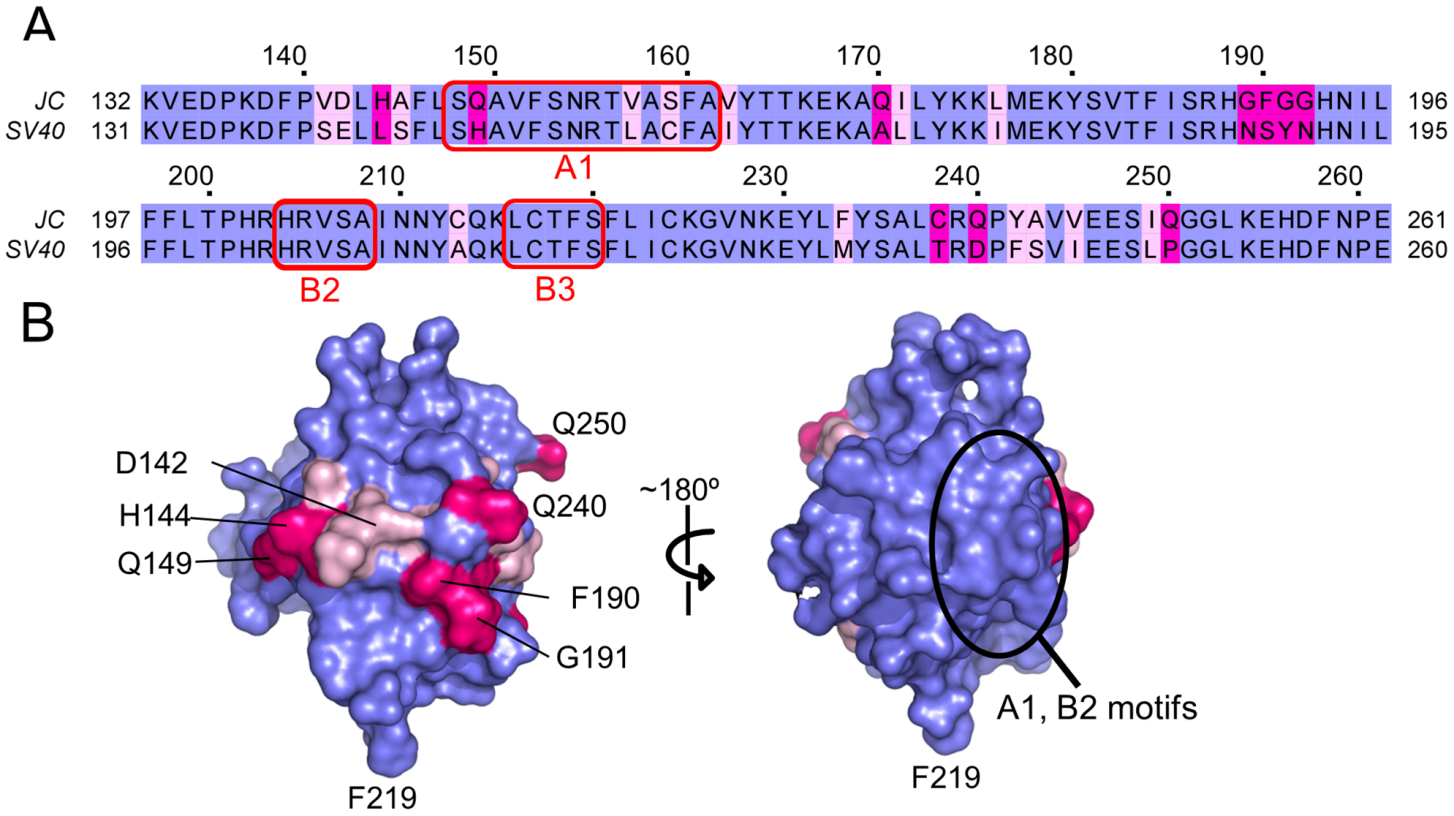
The structure of the JCV T-ag OBD viewed in terms of residues that are identical conserved, or not conserved, with the SV40 T-ag OBD. **A**. A comparison of the amino acid residues found in the OBDs from JCV (top) and SV40 (bottom). Identical residues are shown in blue, conserved residues in light pink and non-conserved residues in magenta. The locations of the DNA binding A1 and B2 loops are indicated as is the B3 motif; a region involved in T-ag oligomerization. The residue numbers for the JCV OBD are indicated above the amino acid sequence. **B**. Distribution of the identical, conserved and non-conserved residues on the two hemispheres of the JCV OBD. A surface representation of the JCV OBD is shown, it uses the coloring scheme described in A. The region containing the A1 and B2 motifs is circled. In addition, to establish the orientation of the domain, certain residues are labeled.

Phylogenetic studies have established that the amino acid sequence for JCV T-ag is very similar to that of SV40 T-ag (e.g., [37]). Indeed, the amino acid sequence identity between the JCV and SV40 OBDs is 81.5% (106 amino acids identical/130 amino acids (Fig. 2A)). Given that the structures of the JCV and SV40 T-ag OBDs have both been determined, it was of interest to analyze these molecules in terms of the distribution of the identical, conserved and non-conserved residues (Fig. 2B; identical (blue), conserved (pale pink), non-conserved (magenta)). As might be predicted, the interior of the molecule is highly conserved as are the A1 and B2 motifs involved in both DNA binding and interface formation (discussed below) (Fig. 2B; right side). The non-conserved residues map primarily to the hemisphere that is opposite to the one containing the A1 and B2 loops (Fig. 2B; left side. Certain of the conserved and non-conserved residues are indicated).

### II. Interactions between the JCV T-ag OBD and DNA Regulatory Regions

**Figure 3:**
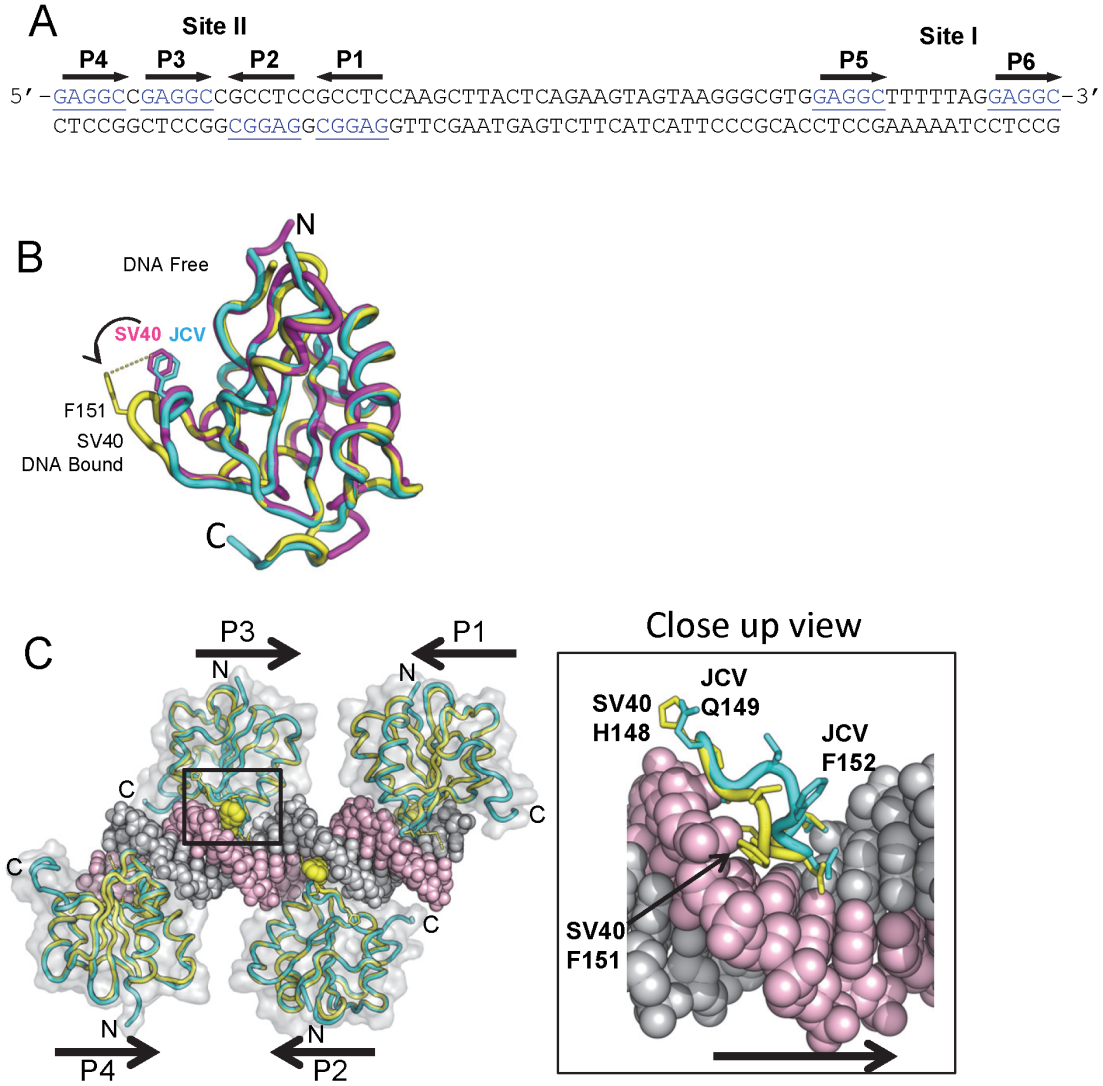
Modeling the interaction of the JCV OBD with the major groove in the Site II region of the core origin. **A**. The DNA sequence of the Site II and Site I regions of the JCV origin of replication. The individucal GAGGC pentanucleotides are indicated. There is a single nucleotide difference between the SV40 and JC virus Site II that was previously shown not to influence T-ag binding [18]. **B**. Comparison of the SV40 OBD structures in the absence and presence of DNA (magenta and yellow structures, respectively) with the DNA free form of the JCV OBD (in cyan). Note the position of SV40 OBD residue F151; based on this superposition, the A1 loop in the JCV OBD structure is in the “apo-conformation.” **C**. Superposition of four JCV OBDs onto the SV40 OBDs bound to the four pentanucleotides in Site II (PDB code 2ITL). A translucent surface representation of the SV40 T-ag OBDs is shown. The locations of the N and C termini are indicated, as are the locations of pentanucleotides P1-P4. Shown as yellow spheres is the position of SV40 T-ag OBD residue F151. The insert shows a close up view of the JCV OBD residue F152 in the major groove and the corresponding residue in the SV40 OBD (i.e., F151) when bound to DNA. This model, indicates that upon interacting with GAGGC sequences in a site-specific manner, the F152 containing A1 motif in the JCV OBD undergoes a structural rearrangement.

The JCV origin of replication contains multiple high affinity GAGGC sequences that serve as binding sites for the JCV T-ag OBD [22]. The GAGGC binding sites are arranged as palindromic repeats in Site II and as direct repeats in Site I (Figure 3A).

#### 1. Interactions of the JCV T-ag OBD on Site II

In view of its extensive homology with the SV40 T-ag OBD, it is apparent that the A1 & B2 loops in the JCV T-ag OBD (Fig. 1C) are needed for site-specific binding to the GAGGC sequences in the JCV origin. Structures of the SV40 OBD, in the presence [42], [47], [49], [63] and absence of DNA [49], [63], established that the A1 loop in the SV40 OBD undergoes a conformational change upon binding to dsDNA (reviewed in [37]). Superposition of the apo (DNA-free) form of the JCV T-ag OBD onto the previously reported SV40 T-ag OBD structures revealed that the A1 region in the JCV T-ag OBD is in the “unbound” conformation (Fig. 3B). We anticipate that a similar conformational change occurs in the JCV T-ag OBD A1 region upon binding to the major groove of GAGGC sequences (Figure 3C; insert). Once this conformation has been adopted, it is likely that the residues in the A1 & B2 regions engage the GAGGC sequences via many of the previously described interactions [42], [47], [49].

**Figure 4:**
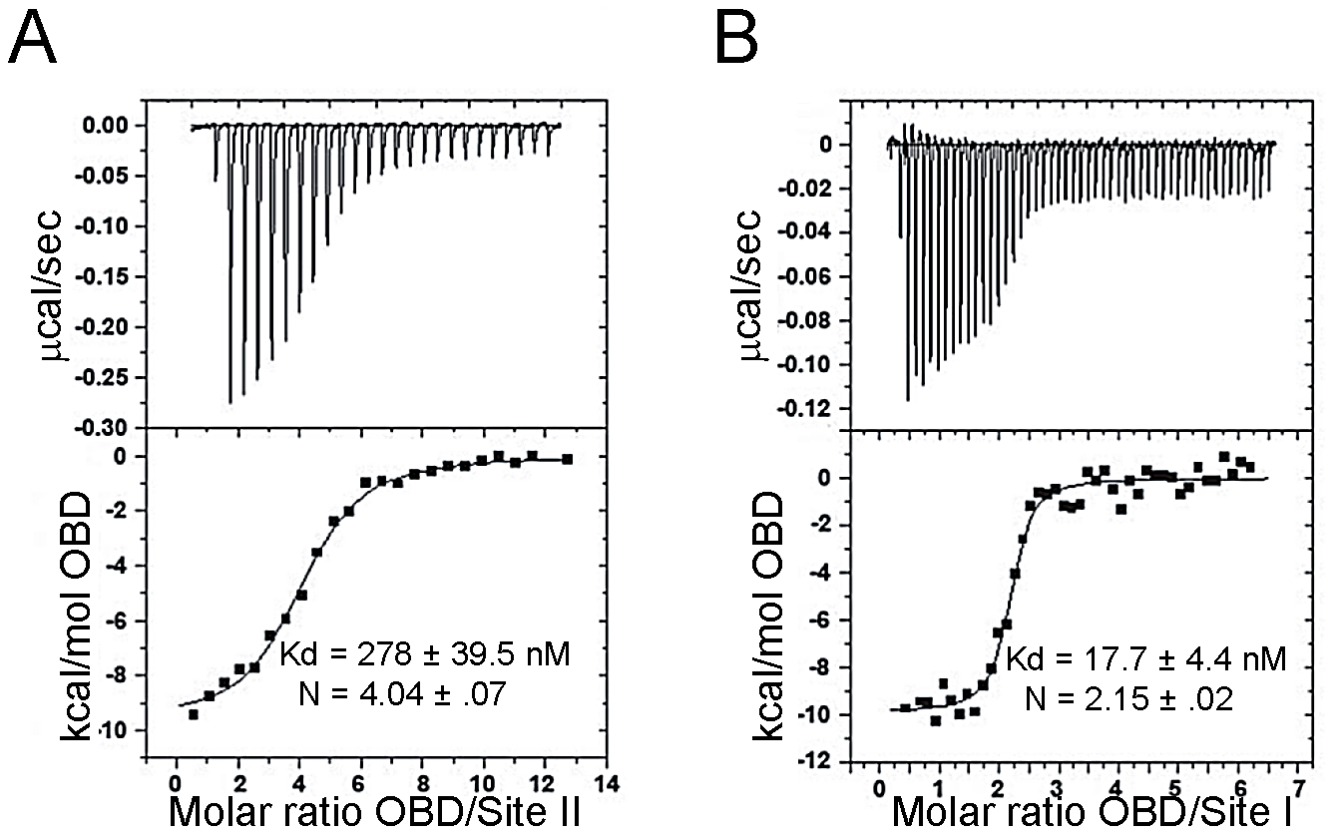
Determining the affinity of the JCV OBD for oligonucleotides containing the Site II and Site I regions of the viral origin. **A**. Results from ITC studies conducted with the JCV T-ag OBD and a 33 bp Site II based oligonucleotide. (The protein concentration in the syringe was ∼80 uM, the Site II containing oligonucleotide was used at a concentration of 1.5 uM). The Site II based oligonucleotide used in this experiment was 5′ TACAGGAGGCCGAGGCCGCCTCCGCCTCCAAGC 3′ and its complement. The calorimetric trace is shown in the top panel; the Kd and stoichiometry (N) values are indicated. The X- axis is time in minutes, while the Y axis of the isotherm is power in ucal/s. These values were determined following curve fitting of the integrated calorimetric trace presented in the bottom panel. **B**. Results from ITC studies conducted with the JCV OBD and a 28 bp Site I oligonucleotide. (The protein concentration in the syringe was 40 uM, the Site II containing oligonucleotide was used at a concentration of 1.0 uM). The Site I based oligonucleotide used in this experiment was 5′ GCGTGGAGGCTTTTTAGGAGGCCAGGGA 3′ and its complement. As in panel A, the calorimetric trace is shown in the top panel; the Kd and stoichiometry (N) values are indicated.

Relative to full-length SV40 T antigen, full-length JCV T-ag binds less efficiently to the Site II and Site I regulatory regions [24], [78]. Therefore, it was of interest to determine if the relatively poor binding of full-length JCV T-ag might be a function of the OBD. To address this issue, we used isothermal titration calorimetry (ITC) to determine the binding affinity and stoichiometry of the components. The duplex oligonucleotides used in these studies are presented in the legend to Fig. 4. The data presented in Fig. 4A demonstrate that four JCV OBDs bind to an oligonucleotide containing the Site II region of the JCV origin with a dissociation constant (K_d_) of ∼278 nM (the average of four titrations was 298.6 nM). This is ∼3 fold weaker than that of the SV40 T-ag OBD bound to an oligonucleotide containing the SV40 Site II DNA target (K_d_ = 93.5 nM when measured by ITC [79]). These studies indicate that relative to SV40 T-ag, the reduced affinity of full-length JCV T-ag for Site II is due, at least in part, to the OBD/DNA interaction.

#### 2. The interaction of the JCV T-ag OBD with Site I

The Site I regulatory region in SV40 is involved in the auto-regulation of early gene transcription (e.g., [80], [81], [82]) and the promotion of DNA replication ([76], [83] and references therein). Site I is also known to stimulate JCV replication (e.g., [19], [29]). Given its importance to JCV replication and other fundamental events, both ITC and modeling studies were conducted to characterize the interaction of the JCV OBD with this region of the JCV origin. ITC studies of the JCV OBD with Site I reveal that the affinity of the JCV OBD for Site I is 18.3 nM (Fig. 4B). Thus, it binds to this site ∼15 fold tighter than to Site II. The SV40 OBD was previously shown to bind SV40 Site I with a Kd of 23 nM [76]. Therefore, the JCV and SV40 OBDs bind Site I with similar affinities and in both cases binding is significantly tighter than to Site II.

**Figure 5:**
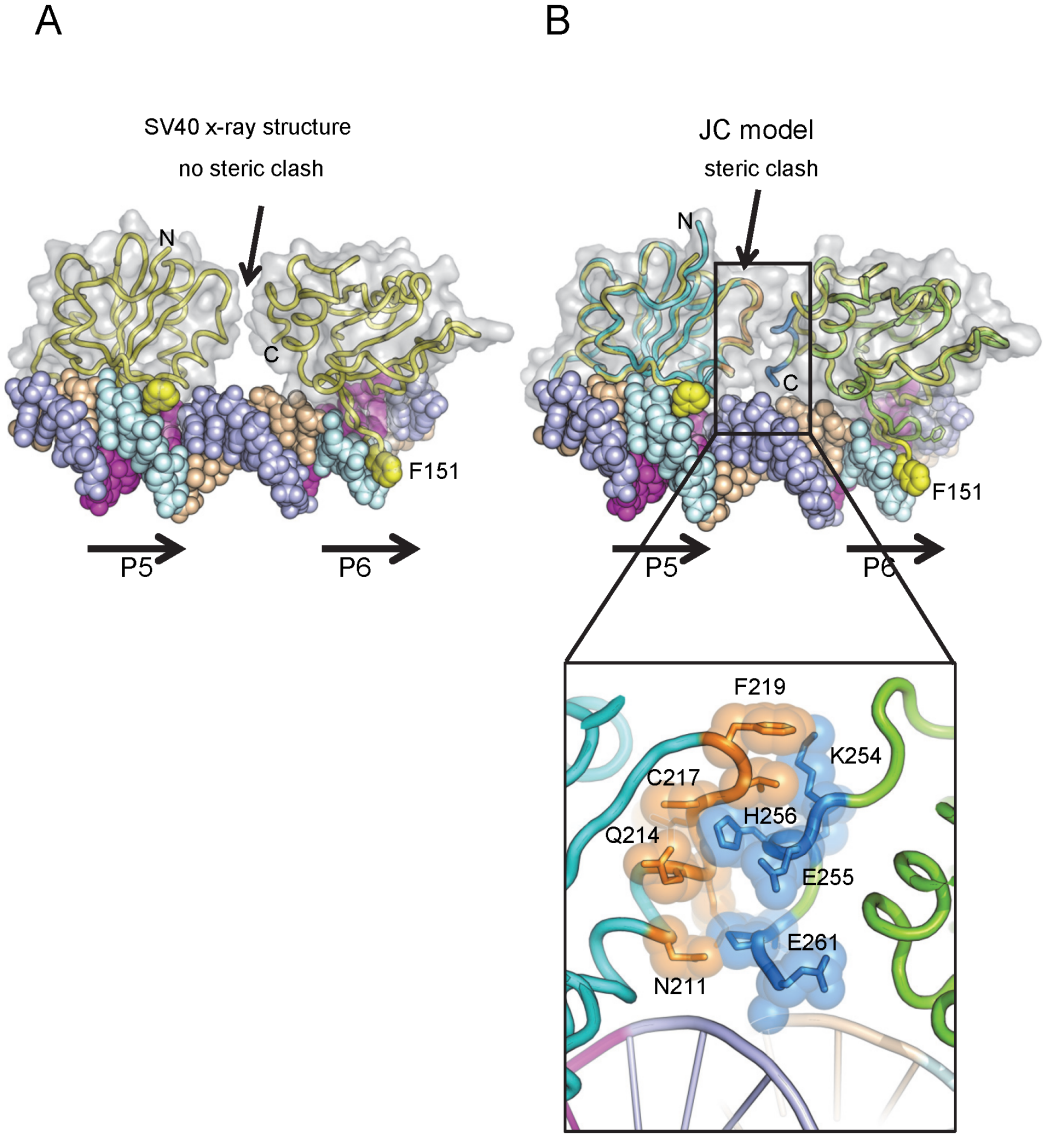
Modeling the interaction of the JCV OBD with Site I. **A**. Presented in panel A is the actual co-structure of the SV40 T-ag OBD on Site I [76]. A translucent surface representation of the SV40 T-ag OBDs is shown, indicating that no protein/protein interactions were observed in this crystal structure. The positions of pentanucleotides P5 and P6 are indicated. Note, in contrast to Site II, the pentanucleotides in Site I are arranged in a head-to-tail orientation [76]. Steric clashes between the OBDs were not detected. The yellow balls indicate the positions of SV40 T-ag OBD residue F151. **B**. A superposition of the JCV OBDs (cyan) onto the SV40 T-ag OBDs (yellow) while bound to Site I (PDB code 4FGN). A translucent surface representation of the JCV T-ag OBDs is presented. Steric clashes occur, an indication that structural rearrangements are likely to take place. Residues that are predicted to clash are shown in the insert.

To explore why this might be the case, we generated a molecular model of the JCV OBD on the JCV Site I sequence. The structure of the SV40 OBD bound to SV40 Site I was recently reported [76]. As in SV40, the two GAGGCs in the JCV Site I are separated by a 7 bp AT-rich sequence. This positions the bound OBDs on the same face of the DNA with a 60 degree angular rotation between the two. In the SV40 T-ag OBD/Site I co-structure, the C-terminus of one OBD was near the B3 region of the second OBD, but no interactions were observed between the OBDs (shown in Fig. 5A). Interestingly, when the JCV OBD was superimposed onto each SV40 OBD in the Site I co-structure (PDB ID = 4FGN) the superposition resulted in minor collisions between the residues at the C-terminus of the JCV OBD bound at P6 and residues near the B3 region of the OBD bound at P5 (Fig. 5B insert). We posit that structural rearrangement of the JCV OBDs and/or the DNA must occur to alleviate these clashes.

### III. Interactions among OBDs

It has been proposed that in the context of a full-length T-ag hexamer, the high local concentration of OBDs promotes their association [42], [84]. To better understand how the JCV T-ag OBDs may assemble during oligomerization, we examined the interactions among the OBDs within the three crystal forms. As described in this section, the largest interface between adjacent molecules is the same in all three crystals. This was unexpected because the three forms belong to different space groups and have different cell dimensions (Table 1).

#### 1. The JCV OBD-OBD interface

**Figure 6:**
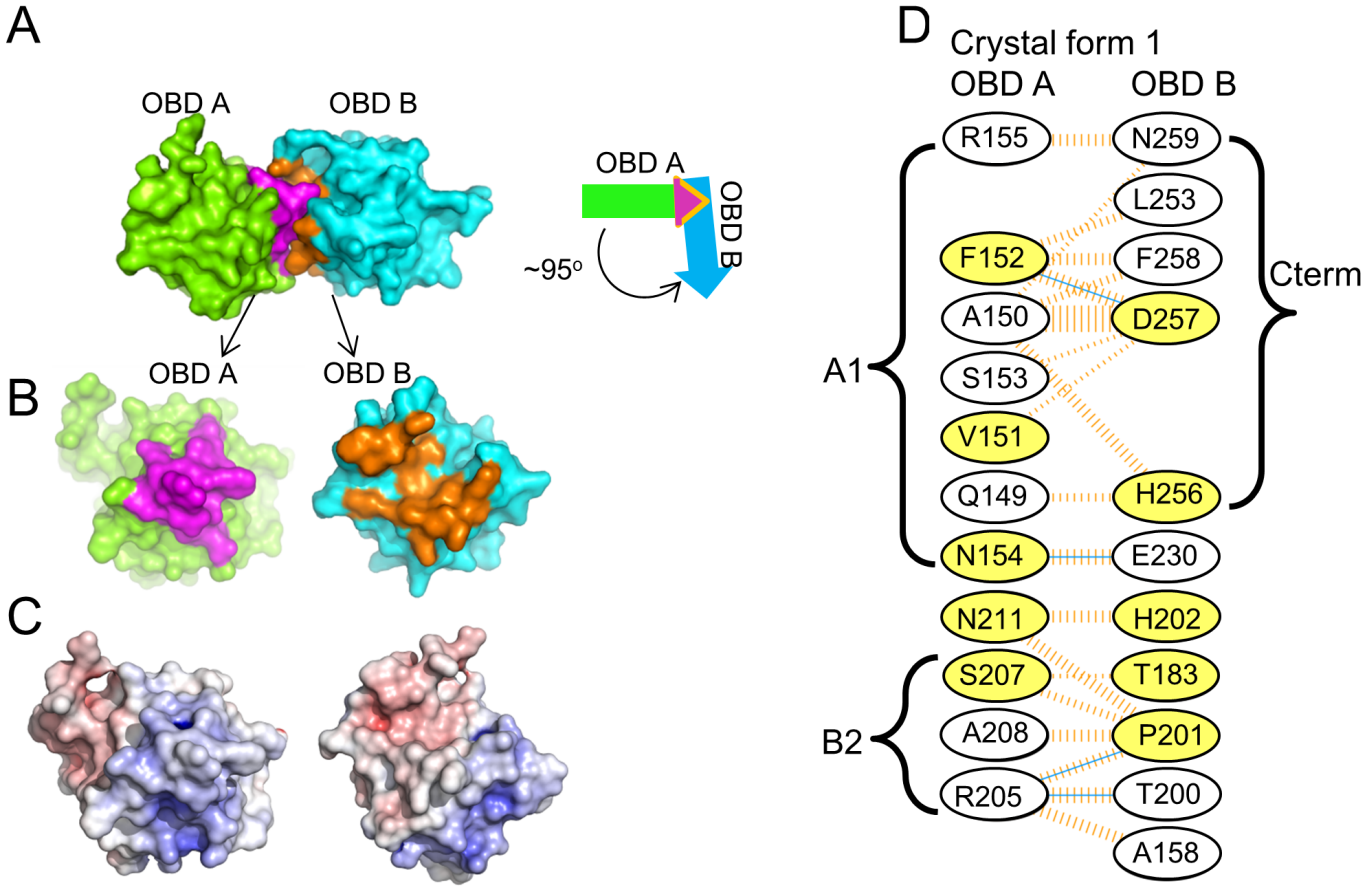
The dimer that represents the asymmetric unit in crystal form 1 of the JCV OBD. **A**. The dimer present in crystal form 1 of the JCV OBD; the positions of monomers A and B are indicated. The purple residues are from the A1 and B2 motifs in monomer A, while the orange residues are from the C-terminal pocket in monomer B. The arrows symbolize that the A and B subunits interact at an approximetly 95 degree angle. **B**. Separation of the dimer to reveal the A1 and B2 motifs (in purple) and the C-terminal pocket (in orange). **C**. The calculated electrostatic potential of the JCV OBD was mapped to the surface of the molecule and color-coded using a sliding scale from −10 to +10 (in units of kB T/e). Red represents negative electrostatic potential, blue positive electrostatic potential and white is neutral. This view of the A1 and B2 motifs and C-terminal pocket emphasizes their electrostatic complementarity. **D**. Residues forming the interface between monomers A and B. The interface includes those from the A1 (i.e., Q149 - R155) and B2 (i.e., R205 - A208) regions of OBD monomer A interacting with residues from monomer B including the C-terminus (i.e., L253 - N259) and those from various loops (e.g., A158, T183, T200 and P201). Hydrogen bonds are indicated by solid blue lines. Non-bonded contacts are indicated by dashed orange lines; the width is proportional to the contribution of the interaction. Finally, those residues that are common to the interfaces formed by the SV40 and JCV OBDs are shaded yellow.

Crystal form I crystallized with two OBDs in the asymmetric unit. The molecules are orientated in a head-to-tail manner and situated at approximately right angles to each other (94.5°; Figure 6A). This dimer contains a small interface (∼550 Å^2^) that is buried between the adjacent JCV OBDs. Residues forming this interface include those from the previously discussed A1 and B2 motifs in one monomer (purple residues in Figs. 6A & B) with C-terminal residues in the second OBD (orange residues in Figs. 6A & B). More specifically, the interface is comprised of residues from the A1 motif (i.e., 149–155) and a face of helix α-C ((residues 205, 207, 208 & 211); which includes a portion of the B2 motif (AA204–208)) fitting snugly into a U-shaped pocket of the second OBD (discussed below). The residues forming the interface are presented in figure 6D; inspection of this figure reveals that these residues form four hydrogen bonds and that there is a significant hydrophobic character in the interaction.

A similar interface was observed in a previous crystal structure of the SV40 T-ag OBD [63], [77]. Indeed, ∼50% of the residues involved in forming the SV40 interface are utilized in the JCV OBD/OBD interface (Fig. 6D; yellow residues). In both instances, the interface is formed when the positively charged A1/B2 motifs on one OBD insert into a negatively charged groove of the second OBD (Fig. 6C). One consequence of this interaction is that the A1 & B2 loops are largely sequestered within the interface, and as a result no longer available for site-specific binding [63], [77]. Finally, the residues used to form the SV40 and JCV T-ag OBD interfaces are highly conserved in other polyomavirus T-ag OBDS (data not shown). Thus, it is hypothesized that the interface formed by the JCV and SV40 T-ag OBDs may be a common feature of other polyomavirus T-ags during hexamer formation.

#### 2. The pocket in the JCV T-ag OBD

**Figure 7:**
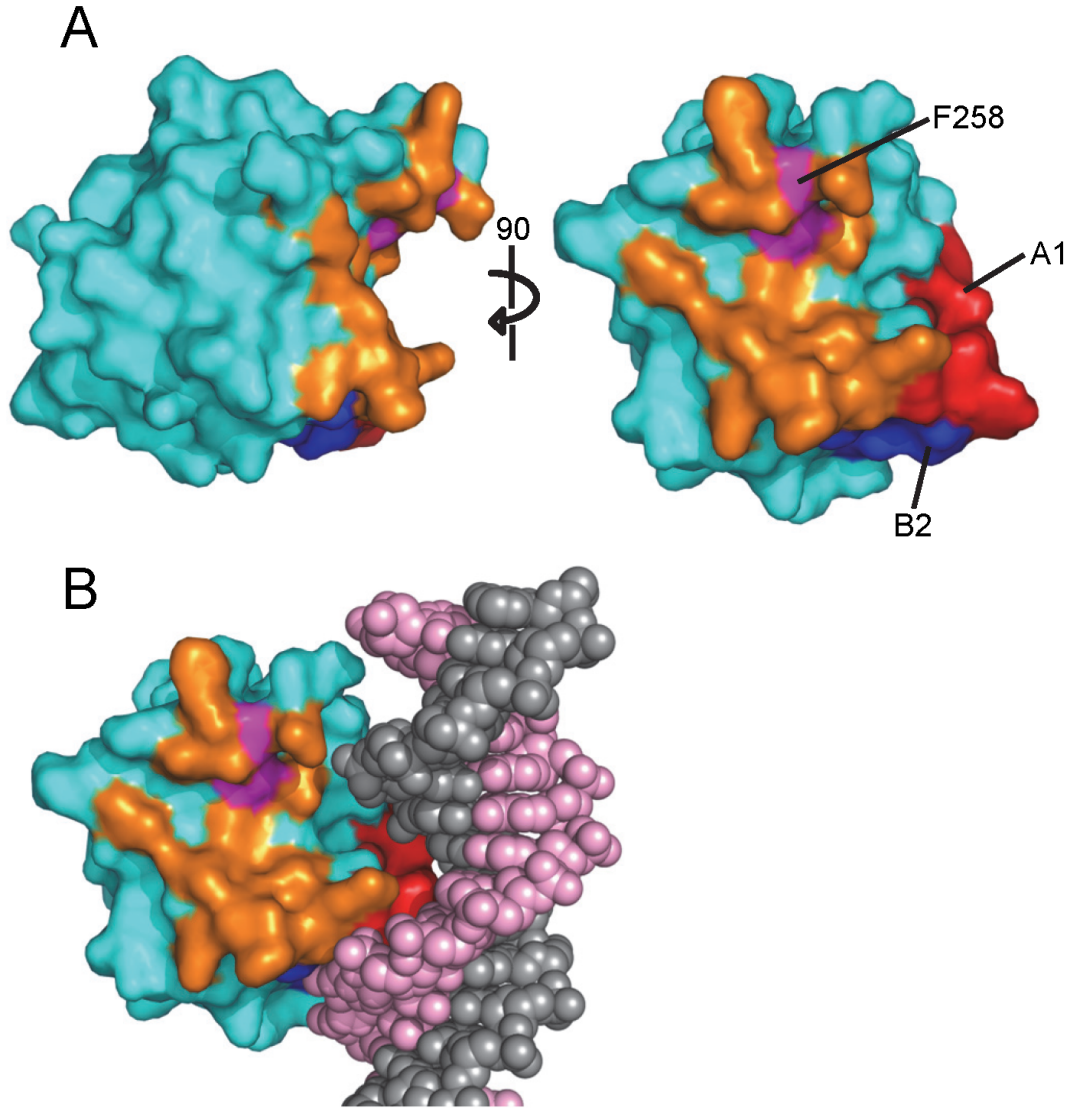
The novel pocket present in the JCV OBD. **A**. Two views of the newly identified pocket present in the JCV OBD; residues lining the pocket are shown in orange. Pocket residue F258 is colored in purple. **B**. A model depicting the relative position of the pocket on a JCV OBD site-specifically bound to the major groove of duplex DNA.

As noted above, the interface between JCV T-ag OBDs involves residues from the A1 and B2 motifs from one molecule and the C-terminal residues of the other (Fig. 6D). The A1 and B2 loops fit snugly into the U-shaped groove or pocket of the second OBD (Figs. 6B and 7A). The relative positions within the JCV T-ag OBD of the pocket and the A1 and B2 loops are presented in Fig. 7A; right). The JCV T-ag OBD pocket is more pronounced than the analogous cleft in previous structures of the SV40 T-ag OBD. Moreover, the pocket in the JCV OBD exhibits both charge and shape complementarity to the A1 and B2 loops (Fig. 6C). Furthermore, a model depicting the location of the pocket on a JCV T-ag OBD molecule site-specifically bound to DNA has been generated (Fig. 7B). This model indicates that when the JCV T-ag OBD is bound to the origin, the pocket is exposed and therefore potentially accessible to the A1 and B2 loops of a second OBD molecule.

#### 3. The common higher order structure observed with the JCV OBDs

**Figure 8:**
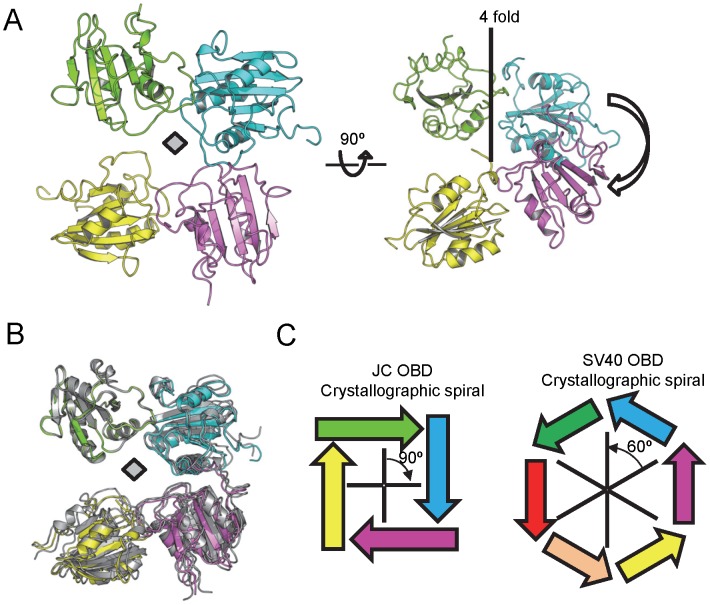
The higher order structures present in the JCV T-ag OBD crystals. **A**. Two views of the right handed tetrameric crystallographic spiral formed by the JCV T-ag OBD. **B**. Superimposition of crystal forms 2 and 3 and their relevant symmetry mates on crystallographic spiral of form 1. The JCV T-ag OBDs in crystal form 1 are colored, while those in crystal forms 2 and 3 are in gray. **C**. Contrasting the structures formed by the JCV and SV40 T-ag OBDs. The JCV OBDs interact at an approximately 90 degree angle, while the SV40 OBDs interact at a 60 degree angle [63]. Regarding the translational component, for the SV40 hexameric spiral the rise is ∼6 Å per OBD pair. In contrast, for the tetrameric JCV OBD structures the rise is ∼9 Å per OBD pair (thus both spirals have an overall rise of 36 Å). Consequences of the greater rise seen in the JCV OBD structure include the smaller central channel and the “tighter” spiral observed in the current structures (Fig. 8A).

Previous studies of the SV40 T-ag OBD established that it forms a left-handed hexameric spiral in the crystal [63], [77]. Additional evidence that the SV40 T-ag OBD forms a spiral has been obtained from EM [85] and modeling studies [76]. Therefore, we examined the higher order structure that the JCV T-ag OBD adopts in crystal form 1. Interestingly, the JCV OBD forms a right-handed tetrameric spiral (Fig. 8A). The interface formed in this crystallographic spiral was presented in figure 6. The angle between the monomers is ∼900 and the rise per monomer is ∼9 Å.

To extend these analyses, we examined the interactions among OBDs within crystal forms 2 and 3. Therefore, crystal forms 2 and 3 were superimposed on one OBD from crystal form 1 and the relevant symmetry mates displayed (Fig. 8B; crystal form 1 is colored as in Fig. 8A, while the other two forms are colored gray). It is apparent from Fig. 8B that in all three crystals, the molecules are orientated in a head-to-tail manner and situated at approximately right angles to each other (Fig. 8B). Furthermore, the near perfect superposition indicates that the interfaces are nearly identical. In addition, crystal forms 2 and 3 also formed a right-handed tetrameric spiral (data not shown). Schematics depicting the crystallographic spirals formed by the JCV T-ag OBD and the SV40 T-ag OBD (PDB entry 2FUF) are presented in Fig. 8C (left and right; respectively).

### IV. JCV Replication Assays Conducted with Mutant Forms of JCV T-ag

**Figure 9:**
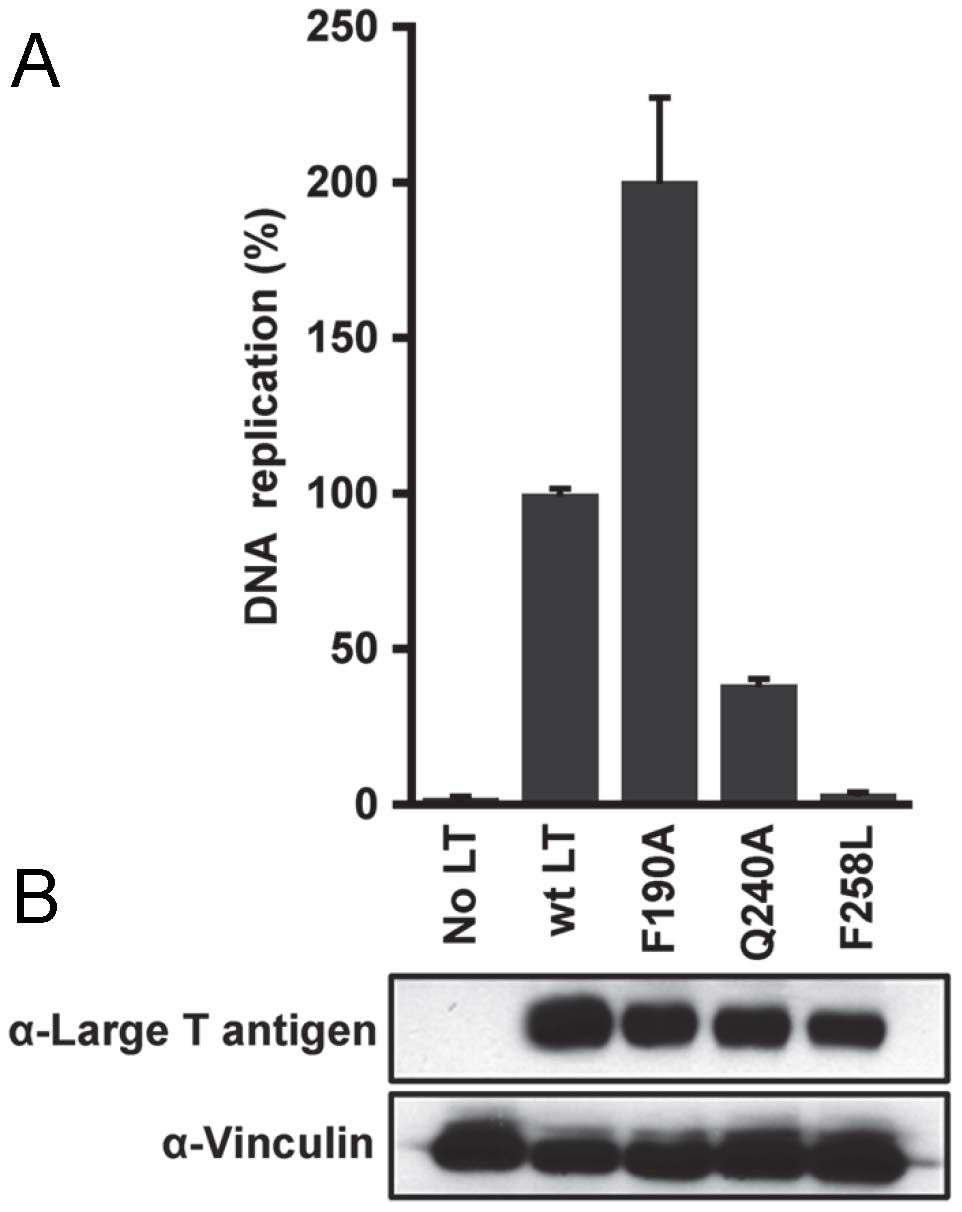
The results of DNA replication studies conducted with full-length JCV T-ag's containing mutations at selected locations. **A**. Relative replication levels of reactions conducted with wild type (wt) JCV T-ag and the Q240A and F258L mutants. The assays (materials and methods) were conducted 72 hrs. post-transfection. **B**. The results of Western Blots used to monitor the levels of the different forms of JCV T-ag in C33A cells at 72 hrs post-transfection. Vinculin levels were determined as a loading control.

In light of the findings derived from our structural studies, it was of interest to determine if particular residues in JCV T-ag are needed for replication. A luciferase-based assay for measuring levels of SV40 and HPV31 DNA replication was previously described [75]. This assay has been adapted for studies of JCV replication using plasmids containing JCV T-ag and the JCV origin of replication (materials and methods). Initially, we used this assay to determine whether residues in the JCV OBD pocket are critical for DNA replication. Inspection of Fig. 9A establishes that a T-ag molecule containing a pocket mutation (i.e., F258L: its location in the pocket is shown in Fig. 7A) does not support DNA replication. Moreover, it is clear from Fig. 9B that the F258L mutation does not cause destabilization of JCV T-ag. (In contrast, two additional mutations in the JCV associated pocket (i.e., L199N and L199R) did cause destabilization (data not shown)). In addition, we initiated studies designed to address whether certain “non-conserved” surface residues (Fig. 2) play a role in JCV replication. Therefore, additional replication assays were conducted with T-ag molecules having the Q240A mutation. Inspection of Fig. 9A establishes that relative to wt JCV T-ag, T-ag molecules containing the Q240A mutation are greatly compromised in terms of their ability to support DNA replication. It is also apparent from Fig. 9B that the decreased ability of the Q240A mutant to support replication is not due to T-ag destabilization. We also analyzed the ability of the F190A mutant to support replication. Surprisingly, this mutant consistently supported higher levels of replication than wild type T-ag (Fig. 9A); a result that is not explained by increased expression of JCV T-ag (Fig. 9B). Finally, no replication of the JCV origin containing plasmid was detected in the control reaction conducted in the absence of T-ag.

## Discussion

The full-length T-ags encoded by both SV40 [86], [87], [88] and JCV [89] form hexamers and double hexamers on their respective origins of replication. Based on previous biochemical and structural studies, we proposed a model for SV40 T-ag's dynamic interactions with the viral origin and its subsequent oligomerization to form double hexamers [37]. One feature of this model is the proposal that following site-specific binding to the GAGGC sequences in the core origin, the OBD domains within SV40 T-antigen rearrange to form hexameric spirals (e.g., [63], [76], [77]). Spiral formation is also a feature of many of the other initiators that have been used as models for studies of the initiation of DNA replication (e.g., [90], [91], [92], [93]). Therefore, spiral formation by replication initiators may be a general phenomenon (reviewed in [94]).

In view of the structures presented herein, we propose that the JCV T-ag OBD undergoes interactions with the JCV origin that are similar to those of the SV40 T-ag OBD (reviewed in [37]). Regarding the initial binding of the OBD to the GAGGC sequences, our analysis of the JCV T-ag OBD structure indicates that the A1 & B2 loops mediate site-specific binding via a mechanism that is similar to that used by the SV40 OBD ([42], [47], [49]; reviewed in [37]). Nevertheless, the ITC studies indicate that there are differences in the interactions between the JCV and SV40 T-ag OBDs and origin sequences. For example, the binding of the JCV T-ag OBD to an oligonucleotide containing the JCV Site II is weaker than the SV40 OBD/Site II interaction [79] (298.6 nM verses 93.5 nM). Related ITC studies demonstrate that the JCV T-ag OBD binds to the GAGGC containing Site I regulatory region with a much higher affinity than Site II (Kds of 18.3 nM and 298.6 nM; respectively. The SV40 T-ag OBD also preferentially bound to Site I [76]). Why the JCV and SV40 T-ag OBDs have different affinities for Site II, and such a wide range of affinities for different GAGGC containing substrates, is not known. Of interest, the B2 regions in the OBDs encoded by JCV and SV40 are identical [40] and there is only one amino acid difference in the A1 regions (H148 in the SV40 OBD is Q149 in the JCV OBD). Therefore, pronounced sequence differences between the A1 & B2 motifs do not explain the observed differences in affinity; however, subtle structural differences in DNA, the OBDs, or both may play a role. Previous SV40 based studies have also established that sequences flanking the individual GAGGC sites play a significant role in modulating OBD binding affinities [95]. Thus, additional studies, including the co-structures of the JCV OBD with oligonucleotides derived from Site II and Site I, are needed to explain the observed differences in OBD affinities for origin sub-fragments. Finally, the full-length T-ag's from JCV & SV40 also have different affinities for Site II [23], [24]. The ITC studies suggest that the differences in the affinities are, at least in part, a function of the OBDs.

The ITC experiments also indicate that four JCV OBDs bind simultaneously to the four GAGGC sequences in Site II. However, in the context of full-length T-ag it is unlikely that all four pentanucleotides are initially bound by the OBDs. This conclusion is based on previous biochemical experiments with SV40 T-ag [96], [97] and structural studies that indicate that once the helicase domain has oligomerized, the shortness of the spacer that links the helicase domain to the OBD restricts OBD binding to only the most proximate pentanucleotide [47]. The subsequent stage(s) during the initiation process at which the initially unbound pentanucleotides are bound by the SV40, and presumably JCV, OBDs remain to be determined. Moreover, studies of both murine [98] and Merkel [79] polyomaviruses have established that in those systems only three pentanucleotide repeats are necessary for DNA replication; further evidence that the interactions of polyomavirus T-ags with the pentanucleotides in Site II are complex.

How polyomavirus T-ags transition from their sequence specific binding mode to fully assembled hexamers and double hexamers is not understood. While the OBDs are monomeric in solution (e.g. [48]), it has been proposed that in the context of T-ag hexamers and double hexamers, the high local concentration of OBDs will promote their association ([42], [84]); reviewed in [37]). Consistent with this possibility, our previous structures of the SV40 T-ag OBD established that it forms a hexameric spiral within the crystal [63], [77]. Therefore, it is of interest that our current studies have established that the JCV T-ag OBD also forms a spiral in the crystal. As in the SV40 T-ag OBD spiral [63], the monomers in the JCV T-ag OBD spiral are arranged in a head-to-tail manner, and the A1 loops are in the DNA-free or “retracted conformation” (reviewed in [37]). An additional common feature of the JCV and SV40 spirals is that they contain a very positively charged central channel that could interact with DNA in a non-sequence specific manner (data not shown). Nevertheless, the spirals formed by the JCV and SV40 T-ag OBDs are not identical. For example, the JCV “spiral” contains 4 OBDs per turn while the SV40 OBD spiral has 6 OBDs/turn (diagrammed in Fig. 8C). In addition, the JCV T-ag OBD forms a right-handed spiral, whereas the SV40 forms a left-handed one. These observations raise the question, “how can different spirals form from T-ag OBDs utilizing very similar interfaces?”

Comparison of the existing spiral structures for the SV40 and JCV T-ag OBDs suggest a common “interface based” model for formation of the observed higher order structures. According to this model, the interface acts like a joint or pivot point and differences in the rotational and translational components of the interface promote the formation of the structures observed to date. For example, in the crystallographic spirals, the angles between the interfaces in the JCV and SV40 T-ag OBDs are very different (i.e., ∼90° and 60°; respectively). In addition, for a spiral to occur, instead of a flat ring structure, there is a requisite translational component (“rise”) to the interface (the SV40 spiral has a rise of ∼6 Å [63], while the rise in the JCV OBD spiral is ∼9 Å). The direction of the translation component relative to the principal rotational axis (i.e., up or down) results in either a left or right-handed spiral (Fig. 8C; legend). Furthermore, in the context of T-ag hexamers and double hexamers, the interactions between the OBDs are likely to be highly dynamic. Support for this postulate includes the relatively small size of the interfaces observed in the crystal structures and previous EM based studies showing multiple orientations of the SV40 T-ag OBDs [85]. In summary, plasticity in the OBD/OBD interface may contribute to the multiple higher-order conformations adopted by the OBD. Nevertheless, it is not known whether the tetrameric JCV T-ag OBD spiral forms *in vivo* or whether it can rearrange into a hexameric OBD spiral that is analogous to the one formed by the SV40 T-ag OBD. However, given the dynamic nature of the domains within T-ag, it is possible that under certain conditions (e.g., following assembly of the hexameric helicase domain), the tetrameric JCV T-ag OBD spiral rearranges to accommodate two additional OBDs.

The C-terminus of the JCV T-ag OBD contains a pocket into which the A1 and B2 residues are inserted. Furthermore, our studies have established that pocket residue F258 is necessary for JCV replication. However, whether this pocket is a general feature of polyomavirus OBDs is not known. The T-ag OBD-DNA co-structures derived from Merkel (PDB entry 3QFQ [79]) and murine polyomavirus (PDB entry 4FB3 [98]) did not contain suitable electron density for tracing of the residues in the C-termini of the OBDs. Therefore no clear pocket was observed in these structures and it is concluded that there is some flexibility in the C-terminal OBD residues. Analyses of SV40 OBD structures revealed that they contain a groove in the same location, but it is not as pronounced as the one in the JCV OBD structure. Regarding evidence for the OBD pocket in larger T-ag structures; a co-structure of a SV40 T-ag dimer, containing both the OBD and the helicase domain (PDB entry 4GDF) interacting with DNA, was recently reported [47]. This structure revealed two completely different orientations of the linker region connecting the two domains. In the structure in which the OBD is bound to pentanucleotide 1, the linker points away from the OBD and the relatively shallower groove is observed. In the second or “hidden site”, the linker bisects the putative pocket. Together, these observations indicate that the “pocket” in SV40 T-ag may be part of a dynamic structure. However, additional structural studies are needed to further characterize the pocket in the SV40 and JCV T-ag OBDs.

Previous studies have also established that the SV40 T-ag OBD serves as a module for binding cellular proteins (reviewed in [38]). For example, the RPA 70AB domain was reported to bind to the T-ag OBD via interactions that include those with R154 [46]. Furthermore, the Nbs1 subunit of the MRN complex binds to the OBD [99]. Given the central roles played by the OBDs during viral DNA replication (reviewed in [37]), the surfaces on the OBDs that interact with these and related cellular replication factors have likely been conserved. Therefore, it is of interest that the JCV and SV40 T-ag OBDs contain one surface that is highly conserved. This surface contains the DNA binding A1 and B2 loops, but also many additional conserved residues that may be involved in binding to cellular proteins (e.g., R154 associated with RPA recruitment). However, it is also apparent that the opposite hemisphere contains the majority of the non-identical residues and certain of these residues (i.e., Q240) are required for JCV replication. These variable regions may simply reflect genetic drift. Alternatively, they may be binding surfaces for cellular proteins encountered in the very different cell types in which these viruses replicate (i.e., monkey kidney cells needed for SV40 replication versus human glial cells needed for JCV replication). Finally, the F190A mutation leads to higher levels of JCV DNA replication. The biochemical basis for this increase is unknown and subsequent studies are needed to address this issue. Nevertheless, a sequence comparison of JCV, SV40 and BK reveals that while JCV T-ag has a bulky aromatic amino acid at position F190, the T-ags from SV40 and BK contain less bulky residues at comparable positions (SV40: S189; BKV: C191). The alanine substitution at JCV T-ag residue F190 introduces an amino acid that requires less space than a phenylalanine. Therefore, the F190A T-ag mutant is more analogous to the SV40 and BKV T-ags and this may be related to the observed increase in DNA replication.

The initiation of JCV DNA replication, and the regulation of this process, is a complicated process. It is apparent that many additional structures will have to be determined before a molecular understanding of the initiation of JCV replication is obtained. Nevertheless, the individual structures of the proteins involved will provide considerable useful information, including potential targets for drug design, such as the pocket within the JCV T-ag OBD described herein.
